# Clopidogrel with Aspirin versus Aspirin Alone following Intravenous Thrombolysis in Minor Stroke: A 1-Year Follow-Up Study

**DOI:** 10.3390/brainsci13010020

**Published:** 2022-12-22

**Authors:** Hai-Ming Cao, Hui-Wen Lian, Yan E, Rui Duan, Jun-Shan Zhou, Xiang-Liang Chen, Teng Jiang

**Affiliations:** 1 Department of Neurology, Nanjing First Hospital, Nanjing Medical University, Nanjing 210006, China; 2 Department of Neurology, Jinling Hospital, Nanjing University School of Medicine, Nanjing 210002, China

**Keywords:** dual antiplatelet therapy, aspirin, minor stroke, thrombolysis, functional improvement

## Abstract

Objective: The objective of this study was to investigate the long-term effect of dual antiplatelet therapy (DAPT) using clopidogrel plus aspirin versus aspirin monotherapy after intravenous thrombolysis on functional outcomes in patients with minor stroke. Methods: Patients with acute ischemic stroke with a National Institutes of Health Stroke Scale score ≤ 5 who received either DAPT or aspirin monotherapy following recombinant tissue plasminogen activator intravenous thrombolysis were studied. Data recorded between January 2017 and December 2020 were retrospectively analyzed. The primary efficacy outcome was functional improvement at 1 year, measured by a 1-point decrease across modified Rankin Scale (mRS) scores. Secondary outcomes included complete rehabilitation (mRS = 0), an excellent outcome (mRS = 0–1), and a favorable outcome (mRS = 0–2) at 1 year, as well as the rates of stroke recurrence and all-cause mortality within 1 year. Results: A total of 238 patients were included, and follow-up data were available for 205 patients (86.1%). The distribution of 1-year outcomes on the mRS favored DAPT over aspirin monotherapy (adjusted common odds ratio (OR), 2.19; 95% confidence interval (CI), 1.12–4.28; *p* = 0.022). Patients who received DAPT, compared with those receiving aspirin alone, were more likely to achieve complete rehabilitation (adjusted OR, 2.44; 95% CI, 1.21–4.95; *p* = 0.013) at the 1-year follow-up. Additionally, the percentages of an excellent outcome and a favorable outcome did not differ, and the rates of stroke recurrence and all-cause mortality were comparable during the 1-year follow-up. Conclusions: Clopidogrel with aspirin following intravenous thrombolysis was associated with improved functional outcome at the 1-year follow-up for patients with minor stroke, and it did not increase the stroke recurrence rate and mortality.

## 1. Introduction

The prevalence of stroke is rising in China [1], and the global burden is ballooning, with a 70.0% increase in the absolute number of incident cases over the last decade [2]. Minor strokes with a low National Institutes of Health Stroke Scale (NIHSS) score represent a substantial number of emergency care visits. According to a recent population-based study, minor strokes account for 40.4% of ischemic stroke hospitalizations [3]. The increase in the use of reperfusion treatment and secondary prevention therapy has helped flatten the disability curve for patients with minor stroke, yet 7.9% of them are disabled or dead by the 1-year follow-up, and this continues to increase to 22.9% by the 5-year follow-up despite mild symptom severity on initial presentation [4]. This poses a considerable challenge in managing patients with minor ischemic stroke, ranging from treatment to prognosis [5].

Dual antiplatelet therapy (DAPT), commonly aspirin and clopidogrel, is recommended by guidelines for patients with minor stroke with non-cardioembolic events who did not receive intravenous thrombolysis [6]. Two independent randomized placebo-controlled trials have established that DAPT started within 12–24 h after symptom onset and continued for 21–90 days can effectively reduce the 90-day risk of recurrent ischemic events in patients with minor stroke [7,8]. The treatment benefit of less recurrent risk has a duration of up to 1 year [9]. Moreover, post hoc analyses have shown an improved functional outcome [10] and reduced stroke-related disability [11] within 90 days. Notably, patients eligible for thrombolysis were not included in the two trials. Hence, it remains open whether the benefits of the combination therapy can be generalized to these candidates. Whilst recent studies have found that DAPT after intravenous thrombolysis improves 90-day prognosis [12,13], the 1-year data on the effectiveness of DAPT in the setting of patients with minor stroke treated with intravenous thrombolysis are lacking.

Despite the clinical significance and research progress, it is worth noting that previous studies have used and are still using various operational definitions of minor stroke. The two most commonly used cut-offs are an NIHSS score ≤ 5 in hyper-acute settings and an NIHSS score ≤ 3 in secondary prevention settings [5]. A large-scale registry has found that NIHSS 4–5 and NIHSS ≤ 3 groups are comparable with regard to in-hospital outcomes [14]; however, whether the long-term prognosis differs significantly in these two groups is unclear.

We aimed to compare the 1-year prognosis of DAPT using clopidogrel plus aspirin versus aspirin alone after intravenous thrombolysis in patients with minor stroke. We hypothesized a positive impact, with the 1-year functional outcomes of the DAPT group improving compared to those of the monotherapy group.

## 2. Methods

### 2.1. Study Participants

This study was based on the stroke registry of Nanjing First Hospital, Nanjing Medical University, a Demonstration Advanced Stroke Center certified by the China Stroke Prevention Project Committee, National Health Commission [15]. It was approved by the Ethics Committee of Nanjing First Hospital, Nanjing Medical University, with the approval number 20211011-05, and it was conducted in accordance with the Declaration of Helsinki. The need for ethical approval and written informed consent was waived by the Ethics Committee of Nanjing First Hospital, Nanjing Medical University, due to the use of a retrospective design and anonymized evaluation of the registry data.

Data from consecutive patients with acute ischemic stroke in the stroke registry recorded between January 2017 and December 2020 were retrospectively analyzed. Studies were eligible for inclusion if the following criteria were fulfilled: (1) 18 years or older; (2) a baseline admission NIHSS score of 5 or less; (3) underwent intravenous thrombolysis with a standard dose of alteplase (Actilyse, Boehringer Ingelheim, Germany) in line with the international guideline [6]; and (4) received DAPT with aspirin (100 mg/day) and clopidogrel (75 mg/day) or aspirin alone (100 mg/day) after 24 h following intravenous thrombolysis for at least 21 days. The exclusion criteria were as follows: (1) a pre-stroke mRS score ≥ 2; (2) a medical history of atrial fibrillation; (3) diagnosed with cardioembolic stroke or stroke of other determined etiology; (4) treated with stent implantation during hospitalization; (5) had hemorrhagic complications after thrombolysis; and (6) other antiplatelet therapy regimens.

### 2.2. Baseline Characteristics

Baseline data on demographics, cardiovascular risk factors, stroke etiology, Oxfordshire Community Stroke Project (OCSP) classification, stroke severity at admission, door-to-needle time (DNT), and combined use of medications were collected through face-to-face interviews by professional neurologists and stroke nurses as part of routine clinical care data in the stroke registry [15]. Cardiovascular risk factors included previous ischemic stroke, transient ischemic attack (TIA), and coronary artery disease (CAD) and a history of hypertension, diabetes mellitus, hypercholesterolemia, and current smoking. Stroke etiologies were classified into three categories: large artery atherosclerosis, small artery occlusion, and stroke of undetermined etiology, which were based on the Trial of ORG 10172 in the Acute Stroke Treatment (TOAST) classification [16].

### 2.3. Outcome Assessments

The primary outcome was functional improvement defined by a 1-point decrease across the modified Rankin Scale (mRS) scores 1 year after the index event. The mRS is an ordinal scale that ranges from 0 (no symptoms) to 6 (death) [17]. The mRS scores were documented within the registry program, as assessed by well-trained neurologists during scheduled interviews by telephone or face-to-face visits. Secondary outcomes included category scores of the mRS at 1 year: 0 (no symptoms at all), 0–1 (excellent outcome, indicating no significant disability despite symptoms), and 0–2 (favorable outcome, indicating functional independence), as well as stroke recurrence and all-cause mortality during the 1-year follow-up period.

### 2.4. Statistical Analysis

Continuous data are presented as median with interquartile ranges (IQRs) and were compared using the Mann–Whitney *U* test. Categorical data are described as frequencies and percentages, with differences being evaluated by using the chi-squared test. The treatment effect using different antiplatelet agents on the primary outcome at 1 year was calculated as an adjusted common odds ratio (OR) for a better distribution of outcomes on the mRS scores; this ratio was estimated with ordinal shift logistic regression. Dichotomized mRS scores at 1 year (0 versus 1–6, 0–1 versus 2–6, and 0–2 versus 3–6) were analyzed using backward stepwise logistic regression, with OR as the effect variable. In these regression models, all effect variables were adjusted for potential imbalances, including the following parameters that were clinically significant or statistically significant in the univariate analysis: age, sex, coronary heart disease, hypertension, diabetes mellitus, stroke etiology, and baseline NIHSS score. All analyses were conducted with SPSS (Version 26.0, IBMCorp., Armonk, NY, USA), and statistical significance was thresholded by a two-sided *p* value of <0.05.

## 3. Results

### 3.1. Study Participants

**Figure 1:**
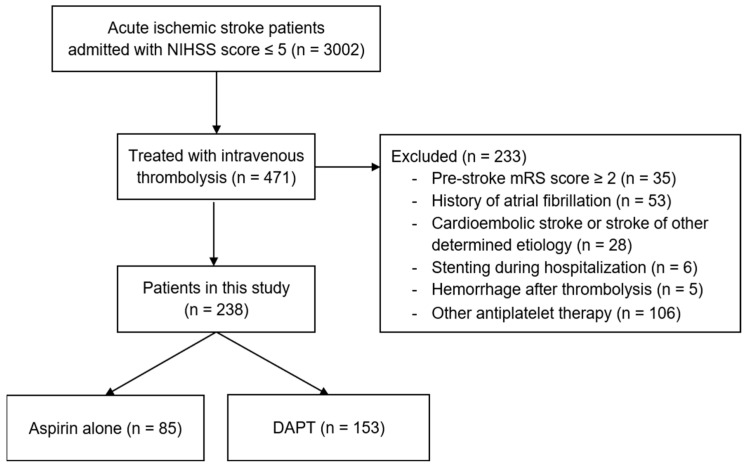
Flow diagram of patient recruitment within the stroke registry data.

From the entire stroke registry of 3002 consecutive patients admitted for acute ischemic stroke with an NIHSS score ≤ 5 between January 2017 and December 2020, 471 patients were treated with intravenous thrombolysis. After the further exclusion of 233 patients (flow diagram shown in Figure 1), 238 patients with a median age of 65 (IQR, 57–73)  years were eligible for analyses in this study, of whom 78.6% were men. The median baseline NIHSS score was 3 (IQR, 2–4), and the median door-to-needle time was 30 (IQR, 20–40) minutes. A total of 33 patients were lost to follow-up: 11 (12.9%) in the aspirin group and 22 (14.4%) in the DAPT group (*p* = 0.758). In total, 205 of the 238 patients (86.1%) had 1-year follow-up data and were included in the analysis of functional outcomes.

### 3.2. Baseline Characteristics

Among the 238 patients included in the study, 85 were in the aspirin monotherapy group, and 153 were in the DAPT group. There was a major difference in the underlying stroke etiology: large artery atherosclerosis (47.7%) was more frequently observed in patients treated with DAPT, whereas strokes due to small artery occlusion (69.4%) were more prevalent in patients treated with aspirin alone. Apart from the history of coronary artery disease that was significantly lower in the aspirin group than in the DAPT group (*p* = 0.016), the other cardiovascular risk factors, namely, previous stroke, TIA, hypertension, diabetes mellitus, hypercholesterolemia, and current smoking, were similar among the groups. The demographic characteristics of age and sex, Oxfordshire Community Stroke Project classification, baseline stroke severity as estimated by the NIHSS score, the door-to-needle time of intravenous thrombolysis, and medications for the 238 included patients were evenly distributed between the two treatment groups at baseline (Table 1).

### 3.3. Primary Outcome

**Figure 2:**
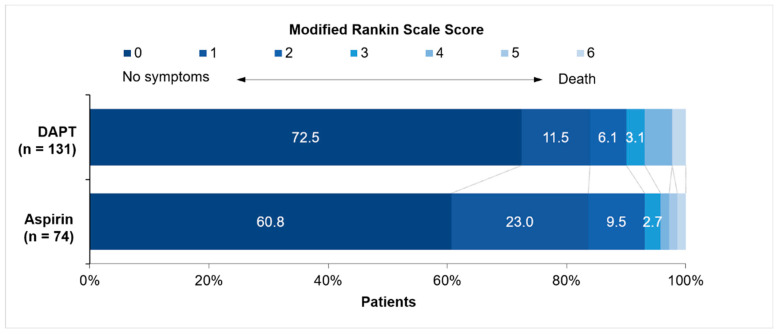
Distribution of modified Rankin Scale scores for patients with minor stroke treated with DAPT and aspirin monotherapy at 1 year.

Of the 204 patients with available follow-up data at 1 year, the adjusted common OR was 2.19 (95% confidence interval (CI), 1.12–4.28; *p* = 0.022), indicating a better distribution of outcomes on the mRS with DAPT than with aspirin alone (Figure 2). The median and IQR of the mRS scores for the two treatment groups and the effect size favoring the DAPT group using univariate and multivariable logistic regression models are shown in Table 2.

### 3.4. Secondary Outcomes

At the 1-year follow-up, the patients in the DAPT group were more likely than the patients in the aspirin group to have no symptoms (i.e., an mRS score of 0) (72.5% vs. 60.8%; adjusted OR, 2.44; 95% CI, 1.21–4.95; *p* = 0.013). No significant differences between the treatment groups were observed among the patients with an excellent outcome (i.e., an mRS score of 0–1) (84.0% vs. 83.8%; adjusted OR, 1.50; 95% CI, 0.63–3.58; *p* = 0.358) and those with a favorable outcome (i.e., an mRS score of 0–2) (90.1% vs. 93.2%; adjusted OR, 0.90; 95% CI, 0.28–2.92; *p* = 0.866) (Table 2). Fourteen recurrent ischemic strokes were reported over the 1-year follow-up period; 11 (8.4%) of the events occurred in the DAPT group, and the remaining 3 (4.1%) events occurred in the aspirin group. The cumulative 1-year rate of death was 2.3% in the DAPT group and 1.4% in the aspirin group, among which one subject died with recurrence. Neither stroke recurrence nor all-cause mortality was significantly different between the two treatment groups (*p* = 0.236 for stroke recurrence rate; Fisher-exact *p* = 0.543 for all-cause mortality).

### 3.5. Subgroup Analyses

The subgroup analyses aimed to determine whether the treatment effect varied across stroke etiologies. For patients with stroke attributed to small vessel occlusion, the treatment with DAPT compared with aspirin alone was associated with higher odds of functional improvement at the 1-year follow-up (adjusted common OR, 4.02; 95% CI, 1.52–10.62; *p* = 0.005), and these patients were more likely to be free from any symptoms (i.e., an mRS score of 0) (82.5% vs. 60.4%; adjusted OR, 4.09; 95% CI, 1.58–10.59; *p* = 0.004). However, for patients who had a stroke due to large artery atherosclerosis, the 1-year functional improvement and dichotomized mRS scores were similar between the two antiplatelet therapy regimens (Table 2).

### 3.6. Outcomes with NIHSS Score ≤ 3 versus NIHSS Scores 4 to 5

The comparisons of the 1-year functional outcomes between admission with an NIHSS score ≤ 3 (n = 140) and admission with an NIHSS score 4–5 (n = 65) showed that the patients with less severe strokes were more likely to be fully rehabilitated without symptoms compared to those with more severe strokes (75.0% vs. 53.8%; *p* = 0.002), and they had a numerically higher proportion of excellent outcomes (i.e., an mRS score of 0–1) (90.0% vs. 70.8%; *p* < 0.001) and favorable outcomes (i.e., an mRS score of 0–2) (94.3% vs. 84.6%; *p* = 0.023) than those with more severe strokes. Moreover, there were fewer events in the NIHSS ≤ 3 group compared with the NIHSS 4–5 group regarding recurrent stroke (5.7% [8/140] vs. 9.2% [6/65]) and death (1.4% [2/140] vs. 3.1% [2/65]), but the differences were not statistically significant (Fisher-exact *p* = 0.380 for stroke recurrence; Fisher-exact *p* = 0.593 for all-cause mortality).

## 4. Discussion

In this retrospective, registry-based, cohort study, we found that patients with minor ischemic stroke who received post-thrombolytic DAPT with aspirin and clopidogrel achieved greater functional improvement at the 1-year follow-up than those receiving aspirin alone following intravenous thrombolysis. The beneficial effect of DAPT was demonstrated by the increased number of subjects with complete rehabilitation, yet DAPT was not associated with a reduced risk of recurrent stroke or mortality within 1 year of stroke onset.

The added value of this study mainly lies in the exploration of the long-term functional outcomes of DAPT in patients with minor ischemic stroke who received intravenous thrombolysis. Our findings suggest a persistent functional improvement for a duration of 1 year in addition to the previously reported 90-day prognostic benefits of DAPT [12,13]. Unlike the dichotomous analysis of the mRS score in prior studies [12,13], we used an ordinal form to assess functional recovery, which has been verified to relate better to long-term outcomes [18]. We also took a dichotomous approach and found a more significant proportion of patients with no symptoms in the DAPT group than in the aspirin group at the 1-year follow-up (72.5% vs. 60.8%). A similar difference was observed in the study conducted by Zhao et al. at 90 days (52.9% vs. 41.0%); however, their percentages of full recoveries were lower. One possible explanation for the higher rate of individuals with no symptoms observed in this 1-year follow-up study is the further rehabilitation beyond 90 days until 1 year after the event, to which a gradient of enhanced sensitivity could extend to even more than 1 year post-stroke [19]. Another attribution might be their inclusion of subjects vulnerable to unfavorable outcomes, including those with a history of atrial fibrillation, those with a history of stroke due to cardioembolism, and those who had hemorrhagic events after thrombolysis [11,20]. Our study excluded these subjects, as they were not eligible for antiplatelet treatment; for instance, anticoagulation rather than antiplatelet therapy is recommended for cardioembolic strokes [6]. Concerning the risk of recurrent stroke and death, the 90-day follow-up studies revealed no statistically significant differences between patients treated with DAPT and patients treated with aspirin [12,13], and our study demonstrated that the event rates remained consistently similar at the 1-year follow-up.

The functional outcomes in the prespecified subgroups showed that the DAPT-related mRS score improvement at the 1-year follow-up did not occur in minor strokes caused by large artery atherosclerosis but in those due to small artery occlusion, primarily demonstrated by a markedly higher percentage of patients who gained complete rehabilitation. This result should be cautiously interpreted due to the limited number of patients in each subgroup. Notably, the stroke etiology was significantly different between the two groups: the DAPT group had a higher rate of large artery atherosclerosis, and the aspirin group had a higher rate of small artery occlusion. This difference could lead to a higher risk of recurrence and mortality in the DAPT group [21,22]. However, our study showed a comparable incidence of stroke recurrence and mortality between the two groups, which suggests that DAPT following thrombolysis might be effective for patients with minor stroke with large artery atherosclerosis. Meanwhile, early investigations testing the efficacy of DAPT for either large or small vessel infarcts have found conflicting results on early neurological deterioration, recurrent ischemic events, and functional outcomes [23,24,25,26]. Hence, there is a need for further evidence to establish the benefit of DAPT for secondary prevention after thrombolysis in minor strokes stratified by different etiologies.

The patients with minor stroke enrolled in our study were defined by an NIHSS score ≤ 5. A recent study comparing different definitions of an NIHSS score ≤ 5 versus an NIHSS score ≤ 3 for minor stroke indicated that the best definition of minor stroke could be extended to an NIHSS score ≤ 5 and that there were comparable short-term composite event rates of death, myocardial infarctions, and recurrent strokes between the NIHSS ≤ 3 and NIHSS 4–5 groups (5.2% vs. 6.6%) [14]. The long-term follow-up findings in our registry showed similar rates of recurrent stroke and mortality 1 year after symptom onset, despite different functional outcomes between the two groups. We believe that these results could enlighten the interpretation of previous studies using different definitions of minor stroke and pave the way for trial comparisons and study design.

Our findings suggest that, at least from the perspective of this study sample, DAPT compared to aspirin alone may improve the long-term prognosis of patients with minor stroke who underwent intravenous thrombolysis. However, several limitations of our study should be noted. Firstly, the single-center observational nature of this study is an inherent limitation. Secondly, the incomplete follow-up data with a dropout rate of 13.9% at 1 year might impact the estimates of treatment effects. However, the rate of loss to follow-up was similar between the two groups, and it has been suggested that <20% loss might be acceptable for validity [27]. Thirdly, bleeding events representing long-term safety outcomes were not routinely followed up in the registry and, thus, were not available for analyses in this study. Fourthly, the unequal sample size could have led to a loss of statistical power [28], although we accommodated the confounding variables. Finally, the subgroup analyses were likely underpowered due to the small sample size. Whether DAPT is more effective than aspirin in post-thrombolytic small vessel occlusive strokes remains to be demonstrated by a randomized controlled trial.

Taken together, DAPT using clopidogrel with aspirin after intravenous thrombolysis for patients with minor stroke can improve functional outcomes at the 1-year follow-up, and it does not increase the stroke recurrence rate and mortality.

## Figures and Tables

**Table 1:** Characteristics of patients with minor stroke treated with aspirin and DAPT after thrombolysis.

Variables	Aspirin	DAPT	*p* Value
	(n = 85)	(n = 153)	
Age, yr, median (IQR)	64 (55–73)	66 (60–74)	0.139
Male sex, n (%)	70 (82.4)	117 (76.5)	0.289
Medical history, n (%)			
Previous stroke or TIA	18 (21.2)	35 (22.9)	0.763
Previous coronary artery disease	1 (1.2)	14 (9.2)	0.016
Hypertension	56 (65.9)	103 (67.3)	0.821
Diabetes mellitus	22 (25.9)	34 (22.2)	0.524
Hypercholesterolemia	2 (2.4)	3 (2.0)	0.587
Current smoking, n (%)	37 (43.5)	67 (43.8)	0.969
Stroke etiology, n (%)			0.002
LAA	21 (24.7)	73 (47.7)	
SAO	59 (69.4)	76 (49.7)	
SUE	5 (5.9)	4 (2.6)	
OCSP classification, n (%)			0.721
TACI	0	1 (0.7%)	
PACI	58 (68.2)	110 (71.9)	
POCI	16 (18.8)	22 (14.4)	
LACI	11 (12.9)	20 (13.1)	
Baseline NIHSS score, median (IQR)	3 (2–4)	3 (2–4)	0.588
DNT, minutes, median (IQR)	30 (20–40)	30 (21–40)	0.917
Medications			
Statins, n (%)	78 (91.8)	147 (96.1)	0.161
Anti-hypertensive agents, n (%)	57 (67.1)	106 (69.3)	0.724
Anti-diabetic drugs, n(%)	29 (34.1)	50 (32.7)	0.821

DAPT, dual antiplatelet therapy; TIA, transient ischemic attack; LAA, large artery atherosclerosis; SAO, small artery occlusion; SUE, stroke of undetermined etiology; OCSP, Oxfordshire Community Stroke Project; TACI, total anterior circulation infarcts; PACI, partial anterior circulation infarcts; POCI, posterior circulation infarcts; LACI, lacunar infarcts; NIHSS, National Institute of Health Stroke Scale; DNT, door-to-needle time.

**Table 2:** Outcomes and treatment effects for patients with minor stroke at 1 year.

Outcomes at 1 Year	Aspirin	DAPT	Unadjusted OR (95% CI)	*p* Value	Adjusted OR (95% CI) *	*p* Value
Total population	n = 74	n = 131				
mRS score, median (IQR)	0 (0–1)	0 (0–1)	1.49 (0.83–2.69)	0.185	2.19 (1.12–4.28)	0.022
mRS score of 0, n (%)	45 (60.8)	95 (72.5)	1.71 (0.93–1.70)	0.085	2.44 (1.21–4.95)	0.013
mRS score of 0–1, n (%)	62 (83.8)	110 (84.0)	1.01 (0.47–2.20)	0.972	1.50 (0.63–3.58)	0.358
mRS score of 0–2, n (%)	69 (93.2)	118 (90.1)	0.66 (0.23–1.92)	0.444	0.90 (0.28–2.92)	0.866
Stroke due to LAA	n = 17	n = 65				
mRS score, median (IQR)	0 (0–2)	0 (0–1.5)	1.27 (0.46–3.54)	0.648	1.55 (0.53–4.47)	0.421
mRS score of 0, n (%)	9 (52.9)	40 (61.5)	1.42 (0.49–4.17)	0.521	1.70 (0.55–5.27)	0.362
mRS score of 0–1, n (%)	12 (70.6)	49 (75.4)	1.28 (0.39–4.18)	0.687	1.41 (0.41–4.4)	0.581
mRS score of 0–2, n (%)	15 (88.2)	55 (84.6)	0.73 (0.15–3.71)	0.708	0.93 (0.16–5.24)	0.931
Stroke due to SAO	n = 53	n = 63				
mRS score, median (IQR)	0 (0–1)	0 (0–0)	2.87 (1.24–6.64)	0.014	4.02 (1.52–10.62)	0.005
mRS score of 0, n (%)	32 (60.4)	52 (82.5)	3.10 (1.32–7.27)	0.009	4.09 (1.58–10.59)	0.004
mRS score of 0–1, n (%)	46 (86.8)	58 (92.1)	1.77 (0.53–5.93)	0.358	2.46 (0.62–9.70)	0.199
mRS score of 0–2, n (%)	50 (94.3)	60 (95.2)	1.20 (0.23–6.21)	0.828	1.43 (0.24–8.48)	0.693

* Adjusted for age, sex, coronary artery disease, hypertension, diabetes mellitus, stroke etiology and baseline NIHSS score. DAPT, dual antiplatelet therapy; mRS, modified Rankin Scale; IQR, interquartile range; OR, odds ratio.

## Data Availability

The anonymized data analyzed during this study are available from the corresponding authors upon reasonable request.
